# Protective effect of TSLP and IL-33 cytokines in ulcerative colitis

**DOI:** 10.1186/s13317-019-0110-z

**Published:** 2019-03-14

**Authors:** Sahar Tahaghoghi-Hajghorbani, Abolghasem Ajami, Saeedeh Ghorbanalipoor, Zahra Hosseini-khah, Saeid Taghiloo, Peyman Khaje-Enayati, Vahid Hosseini

**Affiliations:** 1 Molecular and Cell Biology Research Center, Mazandaran University of Medical Sciences, Sari, Iran; 2 Department of Immunology, School of Medicine, Mazandaran University of Medical Sciences, Sari, Iran; 3 Lübeck Institute of Experimental Dermatology, University of Lübeck, Lübeck, Germany; 4 Department of Molecular Medicine, School of Advanced Technologies in Medicine, Tehran University of Medical Science, Tehran, Iran; 5 Gut and Liver Research Center, Imam Khomeini Hospital, Mazandaran University of Medical Sciences, Sari, Iran

**Keywords:** Inflammatory bowel disease (IBD), Ulcerative colitis, Thymic stromal lymphopoietin (TSLP), IL-33

## Abstract

**Purpose:**

Inflammatory bowel disease (IBD) primarily includes ulcerative colitis (UC) and Crohn’s disease (CD). Thymic stromal lymphopoietin (TSLP) is a cytokine produced by intestinal epithelial cells (IECs) with immunomodulatory properties that plays an important role in the development of regulatory T cell (Treg) responses and tolerance in the gut. On the other hand, IL-33 has been considered as a cytokine with two different properties, inflammatory and anti-inflammatory functions, the latter may play a protective role against chronic intestinal inflammation. In the present study, we investigated the relative gene expression levels of TSLP and IL-33 molecules in ulcerative colitis.

**Methods:**

Patients with clinical symptoms of colitis undergoing a routine diagnostic colonoscopy were included in this study. Biopsy specimens were collected and divided into two parts. One part was fixed and processed for routine histopathological examinations and the other part was stored for RNA extraction. TSLP and IL-33 gene expression were determined using the SYBR Green qRT-PCR.

**Results:**

The expression level of TSLP and IL-33 were significantly lower in UC patients compared with the control group. Moreover, the expressions of these cytokines were more down-regulated in severe UC patients compared with mild and moderate ones and the control group. We also showed a positive correlation between low expression of TSLP and IL-33 and the severity of UC disease.

**Conclusions:**

In this study, we showed decreased mRNA expression levels of TSLP and IL-33 in UC patients and also a negative correlation between expression of TSLP and IL-33 and severity of UC disease.

## Introduction

Ulcerative colitis (UC) is a kind of inflammatory bowel disease (IBD) that characterized by recurrent colonic mucosal inflammation and constitutive dysregulation of cytokine production [1]. Although the etiology of UC remains unclear, it may be related to the genetic and environmental factors, along with dysfunction of the host immune responses against gut microbiota [2] among which, immunological factors are particularly important. Evidence suggest that defects in the apoptotic pathway of T cells and intestinal epithelial cells (IECs) are important in the pathogenesis of the disease. IECs play a crucial role in immune hemostasis through the barrier function as well as innate immune defense and ability to modulate intestinal immune responses [3, 4]. Intestinal epithelial cells produce thymic stromal lymphopoietin (TSLP) cytokine with immunomodulatory properties. IEC-derived TSLP is produced in response to signals received from commensal bacteria which promote dendritic cells (DC) and macrophages with tolerogenic properties. IEC-derived TSLP also contributes to the development and function of regulatory T cell (Treg) responses at mucosal sites [5, 6]. Besides anti-inflammatory and tolerogenic activity, TSLP seems to play a pro-inflammatory role. TSLP activates myeloid DCs and increases the expression of MHCII, CD80, CD83, and CD86 molecules on the surface of these cells. Activated myeloid DCs then stimulate naive T CD4^+^ cells and induce inflammatory T helper 2 (Th2) cells through the development of type 2 cytokines and activation of noncanonical NF-κB signaling. TSLP can also act directly on T cells via promoting proliferation and differentiation of naive CD4^+^ T cells to Th2 cells through the induction of IL-4 gene transcription. TSLP also stimulates cytokine production from mast cells, NKT cells, and eosinophils [7, 8]. Impairment of TSLP production by IECs due to inappropriate DC activation, increasing production of pro-inflammatory cytokines, and development of intestinal inflammation causes intestinal disorders, such as IBD [9–12]. Many studies indicate that TSLP and other cytokines that their receptors share common gamma chain play a main role in regulation the activity of immune cells and are thus important for therapeutic approaches [4, 13–15].

Interleukin-33 (IL-33), a novel identified member of the IL-1 family, has been reported to be produced by several different cell types, such as fibroblasts, adipocytes, endothelial cells, and intestinal epithelial cells [2, 16]. IL-33 plays a major role in improving and controlling the bowel inflammation, particularly in UC [17]. IL-33 has a single domain that binds to its receptor, IL-1 receptor like 1 (IL1RL1), also known as suppression of tumorigenicity 2 (ST2), and eliminates intestinal parasite infections through the induction of Th2 cytokines, such as IL-13 and IL-5 [18–21]. ST2 receptor is expressed on human and mice basophils, eosinophils, mast cells, monocytes, DCs, NKT cells, and Th2 lymphocytes [22–24]. This cytokine induces development of tolerogenic DCs and macrophages and finally leads to induction of iTregs [25]. In some studies, decreased levels of IL-33 and elevated levels of soluble ST2 (sST2), a decoy receptor of IL-33, was observed in sera from patients with IBD compared with healthy individuals [17]. In fact, IL-33 has been considered as a cytokine with dual functions. In the normal conditions, it can promote macrophage polarization towards M2 phenotype and enhance TGF-β expression which is important in the induction of Tregs. In intestinal inflammation, however, IL-33 play its protective role by Th2 induction. The aim of this study was to determine the relative gene expression of TSLP and IL-33 in UC patients and healthy individuals.

## Materials and methods

### Sample collection and processing

In this case–control study, sixty patients with 42.53 ± 3.42 age average and clinical symptoms of colitis who underwent a diagnostic colonoscopy at Tooba Outpatient Clinic (Mazandaran University of Medical Sciences, Sari, Iran) were enrolled in this study. None of the subjects received cyclosporine, non-steroidal anti-inflammatory drugs (NSAIDs), or any antibiotics during past 2 weeks. Patients underwent colonoscopy for the first time. Patients who showed signs of inflammatory bowel disease, such as edematous mucosa, submucosal bleeding, or ulcers, confirmed by a pathologist, were considered as ulcerative colitis, and those without the mentioned signs, were considered as controls. Patients with ulcerative colitis were further divided into three subgroups, including mild, moderate, and severe based on global colonoscopies appearance and clinical signs and disease activity. The biopsy specimens were collected and divided into two parts. One part was fixed and processed for routine histopathological examinations and the other part was stored for RNA extraction. This study was approved by the Ethics Committee of Mazandaran University of Medical Sciences (Ethics Committee Code: Mazums.rec.1391.91-49) and informed consent was obtained from all patients.

### RNA isolation and cDNA synthesis

The biopsy specimens were collected and each tissue specimen was homogenized using mortar and pestle at room temperature. Total RNA was extracted from the dissected tissues using a commercial RNA extraction kit (RNeasy Minikit, Qiagen) according to the manufacturer’s instructions. The quantity and quality of extracted RNAs were assessed by a nanodrop spectrophotometer (Thermo Fisher Scientific Inc., USA) and agarose gel electrophoresis, respectively. RNA (1 μg) was reverse-transcribed into complementary DNA (cDNA) using the RevertAid™ FirstStrand cDNA Synthesis Kit (Fermentas) primed with random hexamer primer in accordance with the manufacturer’s instructions.

### Quantitative reverse transcriptase polymerase chain reaction (qRT-PCR)

The sequences of TSLP and IL-33 along with Glyceraldehyde 3-phosphate dehydrogenase (GAPDH), as a reference gene, were obtained from the GenBank (Table 1). Quantitative PCR was performed using SYBR Green PCR and primers for amplification of TSLP, IL-33and GAPDH were designed using the Beacon designer 7 software and synthesized by TIBmol (Germany) (Table 1).

**Table 1:** Primers and probes used for qRT-PCR quantification

Gene	Accession number	Primers and Probes (5′–3′)	Product size (bp)
TSLP	*XM_011543698.1*	F: 5′-TCG GAA ACT CAG ATA AAT GCT AC-3′	115
		R: 5′-TGA AGC GAC GCC ACA ATC-3′	
IL-33	*XM_017015285.1*	F: 5′-TGC ATG CCA ACA ACA AGG AAC-3′	133
		R: 5′-ACT CCA GGA TCA GTC TTG CAT TC-3′	
GAPDH	*NM_002046.5*	F: 5′-GCT GGG GCT CAT TTG CAG-3′	135
		R: 5′-GCA GGA GGC ATT GCT GAT G-3′	

F, forward primer; R, reverse primer

Table 1 shows primer sequences of target and housekeeping genes. The housekeeping gene glyceraldehyde-3phosphate dehydrogenase (GAPDH) was used as an endogenous reference gene. All tests were performed in triplicate. qRT-PCR was performed in a 25 µl reaction containing; 15 µl SYBR Premix EX TaqII (2×) (Takara, Japan) 50 ng of total RNA input and 0.05 µM SYBR-Green primer sets. qRT-PCR was performed under the following conditions: 94 °C, 2 min, followed by 94 °C, 30 s; 58 °C, 30 s, 72 °C, 45 s and cycled 40 times. GAPDH allows normalization of the expression level of the target gene (TSLP and IL-33) to the amount of input cDNA. ΔCT between the GAPDH and target genes (CT_Target gene_ − CT_Reference gene_) was calculated for case and control group, and ΔΔCT was calculated by subtracting the average ΔCT of case samples from the average ΔCT of control samples. Fold change expression was carried out using the 2^−ΔΔCT^ method.

### Statistical analysis

Statistical analyses were performed using the SPSS version 17.0 software. Distribution of data was evaluated using Kolmogorov–Smirnov test. Quantitative variables were assessed using independent *t* test or one-way analysis of variance (ANOVA). Qualitative variables were assessed applying χ^2^ or Fisher exact test. Multivariate logistic regression analysis was used to assess any association in the gene expression between the patients and the controls.

Differences between groups were considered statistically significant when P < 0.05.

## Results

### Clinical characteristics of study population

Clinical characteristics of sixty subjects, including 30 UC patients and 30 non-UC subjects, as controls, were shown in Table 2. According to Mayo scoring system, four UC patients (20%) showed mild activity, thirteen (65%) moderate activity and three (15%) severe activity. All subjects in the control group showed normal activity (Table 2).

**Table 2:** Clinical characteristics of subjects

Clinical characteristics	Ulcerative colitis (UC)	Non-ulcerative colitis (non-UC)
Male	40% (8)	50% (11)
Female	60% (12)	45% (9)
Age (less than 40)	50% (10)	10% (2)
Age (more than 40)	30% (6)	35% (7)
Disease duration (less than 1 year)	50% (10)	45% (9)
Disease duration (more than 1 year)	35% (7)	15% (3)
Disease activity (severe)	5% (3)	–
Disease activity (moderate)	65% (13)	–
Disease activity (mild)	20% (4)	–
Healthy	–	100% (20)

### Expressions of TSLP and IL-33in UC patients and healthy controls

**Fig. 1:**
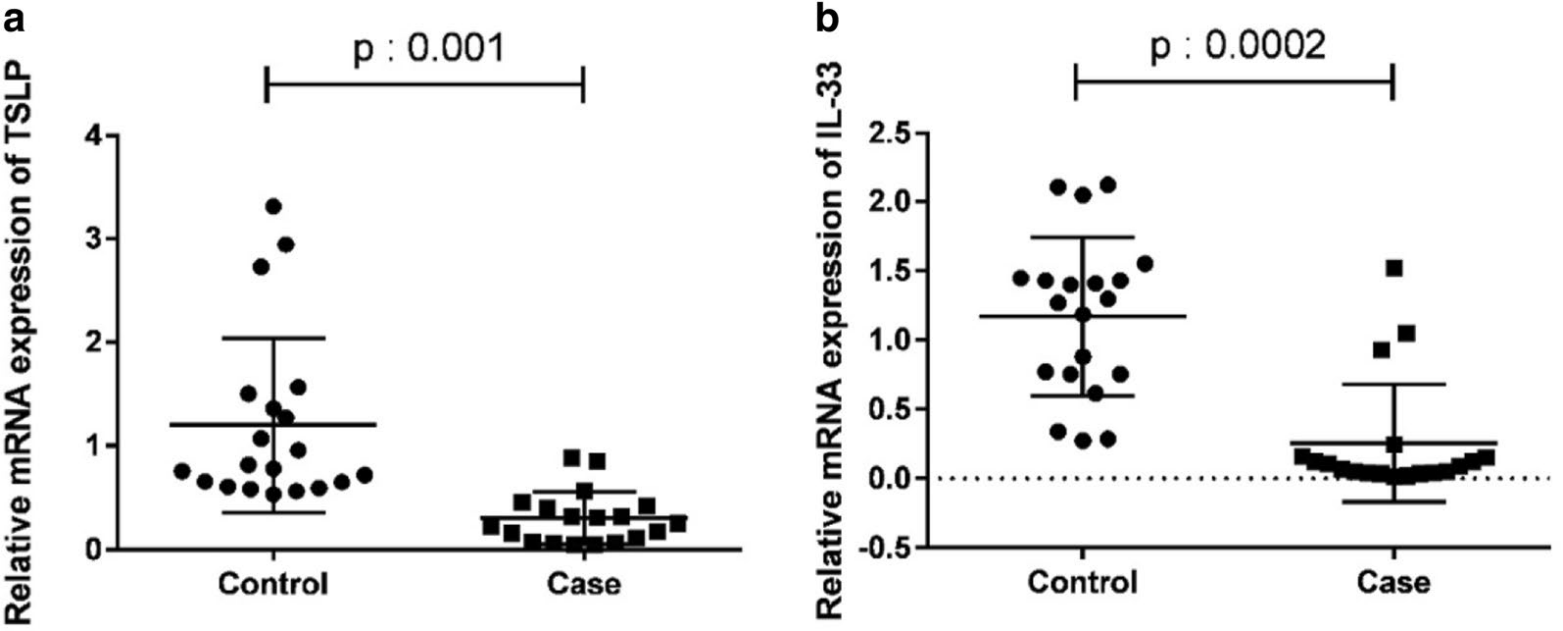
Expression of TSLP and IL-33 mRNA in UC patients and healthy controls. Colonic biopsies were taken from UC patients and non UC subjects. The expression of TSLP mRNA and IL-33 mRNA analyzed by quantitative SYBR Green PCR. The results represent mean ± standard deviation (SD). The expression of TSLP mRNA (**a**) and IL-33 mRNA (**b**) were lower in UC patients compared with control group (P: 0.001 and P: 0.0002, respectively)

To evaluate the relative mRNA expressions of TSLP and IL-33, colonic biopsies from UC patients (N = 30) and controls (N = 30) were analyzed by qRT-PCR. The results showed that the expression levels of both TSLP and IL-33 were significantly lower in UC patients than in healthy subjects (P = 0.001 and P = 0.002, respectively) (Fig. 1a, b).

### Correlation of IL-33 and TSLP expression with severity of disease

**Fig. 2:**
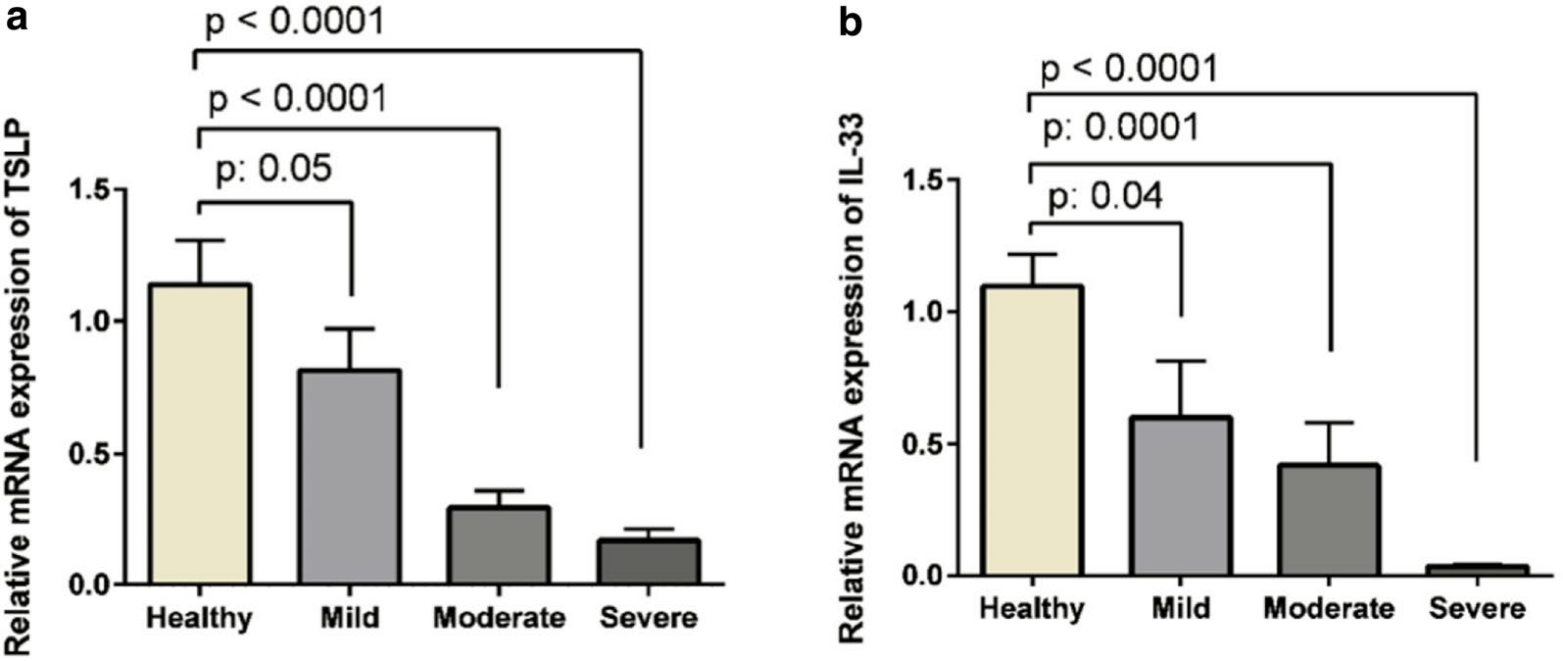
Comparison between the expressions of TSLP (**a**) and IL-33 (**b**) and severity of disease. The relative expressions of TSLP mRNA is down-regulated in severe UC patients compared to mild and moderate ones and control group, respectively

Relative mRNA expressions of TSLP and IL-33 were down-regulated in severe UC patients, mild, moderate and control group, respectively. The expression of TSLP was down-regulated in severe UC patients compared with mild and moderate (Fig. 2a). In addition, the expression of IL-33 was down-regulated in severe UC patients compared with mild and moderate (P = 0.003) (Fig. 2b).

### Comparing between the expressions of IL-33 and TSLP with gender

**Fig. 3:**
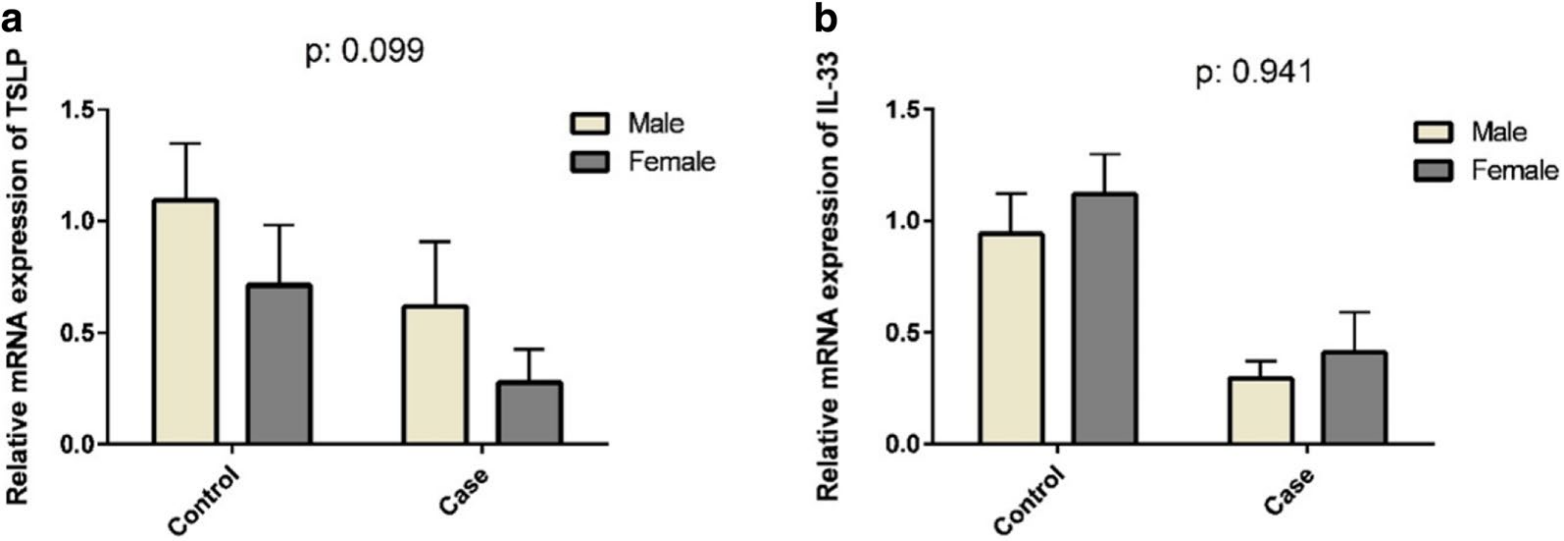
Comparison between the expressions of TSLP and IL-33 and gender. The relative expressions of TSLP mRNA in female were less than male in UC patients and healthy control (**a**) (P = 0.099). The relative expressions of IL-33 mRNA in female more than male in UC patients and healthy control (**b**) (P = 0.941P). The differences were not statistically significant

Expression levels of TSLP in female subjects were lower than in male in both UC patients and healthy controls (P = 0.099) (Fig. 3a), while expression levels of IL-33 in female were higher than in male in UC patients and healthy controls (P = 0.941) (Fig. 3b). They were not statistically significant.

### Comparing between the expressions of IL-33 and TSLP with age

**Fig. 4:**
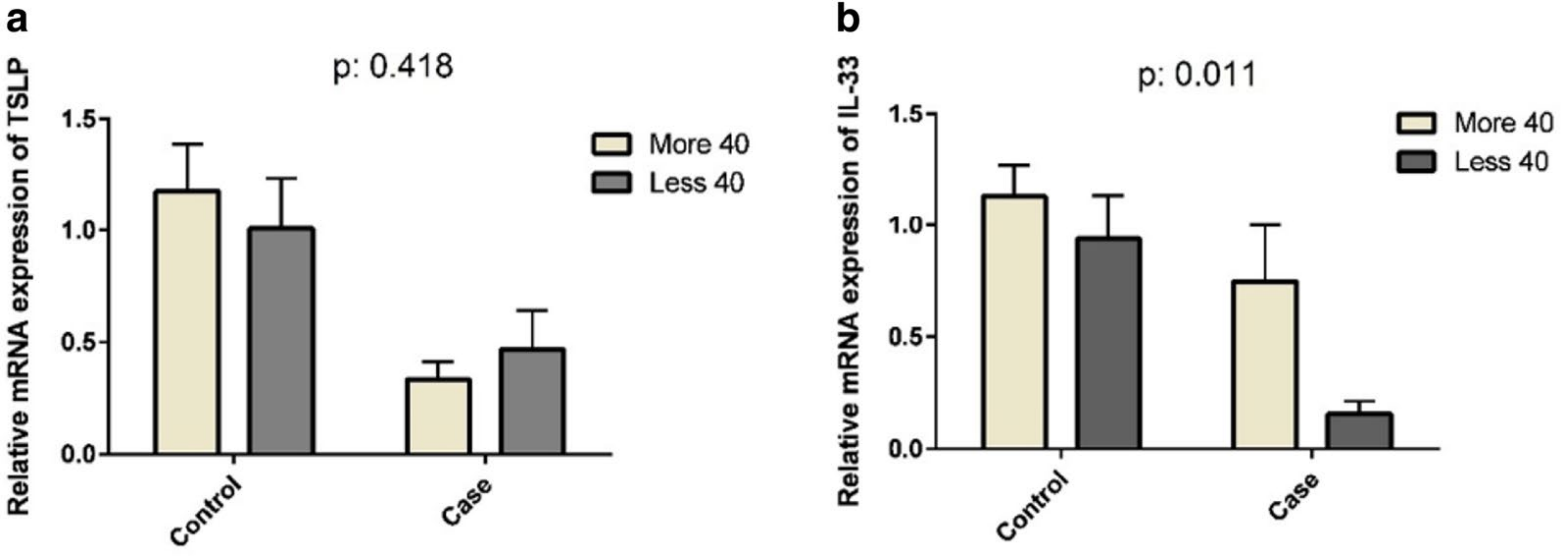
Comparison between the expressions of TSLP and IL-33 with age. The relative expressions of TSLP mRNA (**a**) and IL-33 mRNA (**b**) in subjects over 40 years old less than subjects under 40 years old in UC patients (P: 0.418 and P: 0.011, respectively). They were not statistically significant

TSLP was expressed less in subjects over 40 years old than in those under 40 years old in UC patients (P = 0.418) (Fig. 4a); however, IL-33 was expressed more in subjects over 40 years old than in those under 40 years old in UC patients and healthy controls (P = 0.011) (Fig. 4b).

### Comparison of the expressions of TSLP and IL-33 with stages of disease

**Fig. 5:**
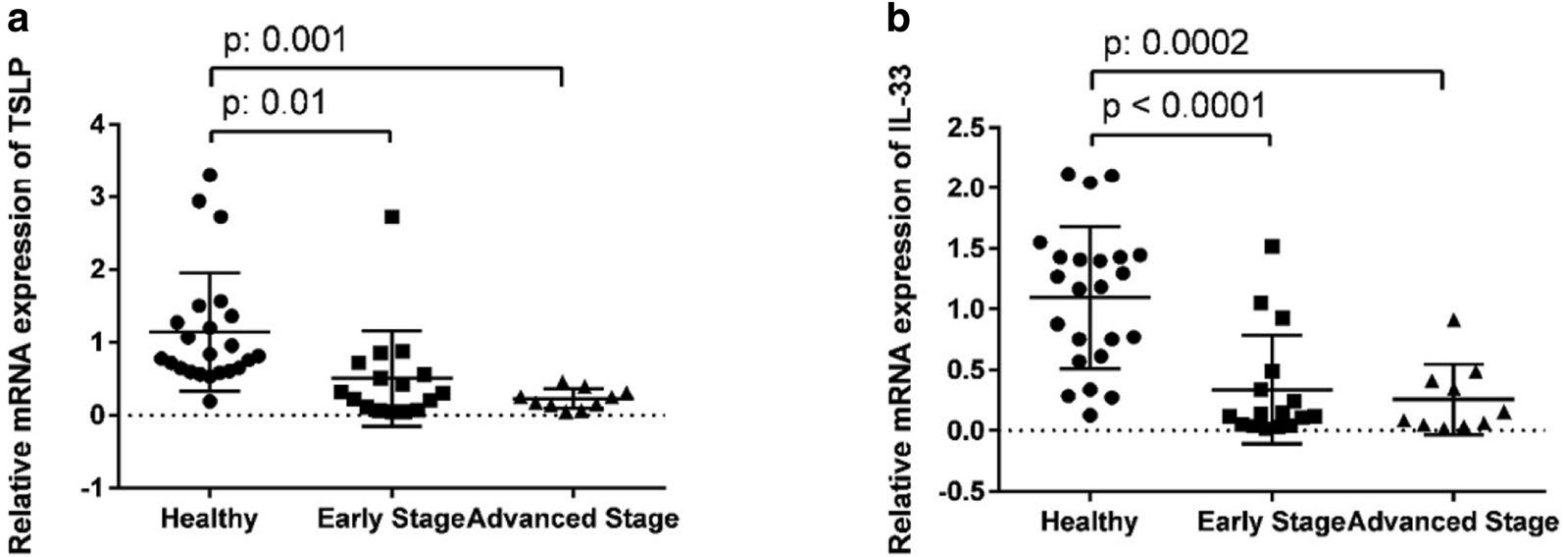
Comparison between the expressions of TSLP and IL-33 with stages of disease. The relative expressions of TSLP mRNA (**a**) and IL-33 mRNA (**b**) in UC patients with advanced stage of disease less than subjects with early stage of disease and healthy controls (P: 0.006 and P: 0.001, respectively)

The expressions of TSLP in UC patients with advanced stage of disease were less than subjects with early stage of disease and healthy control (P = 0.006) (Fig. 5a). Also, the expressions of IL-33 in UC patients with advanced stage of disease less than subjects with early stage of disease and healthy controls (P = 0.001) (Fig. 5b).

## Discussion

In this study, we evaluated mRNA expression levels of IL-33 and TSLP and revealed the low expression of these cytokines in UC patients. We found that the expressions of these cytokines were more down-regulated in severe UC patients compared with mild, moderate ones and the control group. Although the role of IL-33 in IBD has not been completely understood, IL-33 has been known as a cytokine with both pro- and anti-inflammatory properties [26–28]. These dual functions can be resulted from different concentrations of IL-33 [28]. In the optimal situation, intestinal epithelial cells produce IL-33 and TSLP, both induce the development of tolerogenic DCs, M2 macrophages and eventually induction of iTregs. Consequently, macrophages and iTregs produce IL-10 and TGF-β, respectively.

This occurs in the bowel under physiological circumstances and plays a principle role in inhibition of chronic inflammation observed in IBD. In addition, IL-33 induces iTregs in the bowel resulting in healing the inflammatory lesions [17]. IL-33 and TSLP also induce inflammation via eosinophil and mast cell activation by secretion of IL-13 and IL-5. These cytokines deviate the immune responses toward Th2 that have protective effect on UC. Based on our results, expression levels of both IL-33 and TSLP showed a negative correlation with the severity of UC. This shows that high mRNAs expression of IL-33 and TSLP might play a protective role in development and severity of UC. High levels of IL-33 could restore the goblet cells and promote switching of M1 macrophages to M2 phenotype with anti-inflammatory properties which might play healing roles in IBD. On the other hand, low levels of IL-33 fail to promote M1 macrophages to M2 ones and also Th2 induction. This leads to severe forms of UC. In a similar way, the levels of TSLP are correlated with development and severity of disease [28].

There are some compatible and incompatible results from other studies in comparison with our results. Seo et al. showed decreased levels of IL-33 in sera of patients with IBD compared with healthy individuals suggested that IL-33 can attenuate UC. Also their results are consistent with our findings which low levels of IL-33 were correlated with severity of diseases. They also showed the elevated levels of soluble ST2 (sST2), as a decoy receptor of IL-33, in chronic intestinal inflammations [28]. In contrast, Maha et al. showed a significant increase in serum levels of IL-33 in patients with UC and chronic intestinal inflammation compared with healthy control group [2] and Pastorelli et al. showed elevated levels of IL-33 in sera from IBD patients [23]. Also, Kobori et al. showed elevated mRNA expression of IL-33 in active lesions from UC patients [29].

Our results also showed lower mRNA expression of TSLP in UC patients compared to controls. TSLP can promote both Treg and Th2 responses thus inhibit Th1 and Th17 responses that consequently suppress inflammation [12, 17, 30]. Therefore, there are two implications about the properties of TSLP and IL-33. Firstly, these cytokines show anti-inflammatory role via induction of tolerogenic DCs and M2 macrophages leading to development of Treg responses [4, 31, 32]. Secondly, these two cytokines modulate the differentiation of Th cells to promote Th2 cells [12]. Therefore, TSLP and IL-33 play a critical role in intestinal immune homeostasis. Our results support the hypothesis that TSLP and IL-33 have a protective effect in UC and low levels of these cytokines can promote the severity of disease.

## Conclusions

In this study, we showed decreased mRNA expression levels of TSLP and IL-33 in UC patients and also, a negative correlation between expression of TSLP and IL-33 and severity of UC disease. Regarding the Th2 and Treg induction by TSLP and IL-33, it is predictable that IBD will be the consequence of decreased levels of these cytokines.

## References

[1] MacDonald TT, Monteleone I, Fantini MC, Monteleone G (2011). Regulation of homeostasis and inflammation in the intestine. Gastroenterology.

[2] Nazzal MF, Hazima MK, Al-AbassiAli H, Ad’hiah (2014). Investigating the role of il31 in inflammatory bowel disease. Int J Curr Res.

[3] Peterson LW, Artis D (2014). Intestinal epithelial cells: regulators of barrier function and immune homeostasis. Nat Rev Immunol.

[4] Taylor BC, Zaph C, Troy AE, Du Y, Guild KJ, Comeau MR, Artis D (2009). TSLP regulates intestinal immunity and inflammation in mouse models of helminth infection and colitis. J Exp Med.

[5] Rutella S, Locatelli F (2011). Intestinal dendritic cells in the pathogenesis of inflammatory bowel disease. World J Gastroenterol.

[6] Tezuka H, Ohteki T (2010). Regulation of intestinal homeostasis by dendritic cells. Immunol Rev.

[7] Maloy KJ, Powrie F (2011). Intestinal homeostasis and its breakdown in inflammatory bowel disease. Nature.

[8] Pott J, Hornef M (2012). Innate immune signalling at the intestinal epithelium in homeostasis and disease. EMBO Rep.

[9] Abraham C, Medzhitov R (2011). Interactions between the host innate immune system and microbes in inflammatory bowel disease. Gastroenterology.

[10] Boden EK, Snapper SB (2008). Regulatory T cells in inflammatory bowel disease. Curr Opin Gastroenterol.

[11] Ordonez F, Lacaille F, Canioni D, Talbotec C, Fournet JC, Cerf-Bensussan N, Goulet O, Schmitz J, Ruemmele FM (2012). Pediatric ulcerative colitis associated with autoimmune diseases: a distinct form of inflammatory bowel disease?. Inflamm Bowel Dis.

[12] Ziegler SF, Roan F, Bell BD, Stoklasek TA, Kitajima M, Han H (2013). The biology of thymic stromal lymphopoietin (TSLP). Adv Pharmacol (San Diego, Calif).

[13] Liu YJ (2009). TSLP in epithelial cell and dendritic cell cross talk. Adv Immunol.

[14] Rochman Y, Spolski R, Leonard WJ (2009). New insights into the regulation of T cells by γc family cytokines. Nat Rev Immunol.

[15] Soumelis V, Liu Y-J (2004). Human thymic stromal lymphopoietin: a novel epithelial cell-derived cytokine and a potential key player in the induction of allergic inflammation. Springer seminars in immunopathology.

[16] Pastorelli L, De Salvo C, Cominelli MA, Vecchi M, Pizarro TT (2011). Novel cytokine signaling pathways in inflammatory bowel disease: insight into the dichotomous functions of IL-33 during chronic intestinal inflammation. Ther Adv Gastroenterol.

[17] De Souza HS (2016). Immunopathogenesis of IBD: current state of the art. Nat Rev Gastroenterol Hepatol.

[18] Ayako K, Yagi Y, Imaeda H, Ban H, Bamba S, Tsujikawa T, Saito Y, Fujiyama Y, Andoh A (2010). Interleukin-33 expression is specifically enhanced in inflamed mucosa of ulcerative colitis. J Gastroenterol.

[19] Nunes T, Bernardazzi C, de Souza HS (2014). Interleukin-33 and inflammatory bowel diseases: lessons from human studies. Mediat Inflam.

[20] Pastorelli L, De Salvo C, Vecchi M, Pizarro TT (2013). The role of IL-33 in gut mucosal inflammation. Mediat Inflamm.

[21] Pichery M, Mirey E, Mercier P, Lefrancais E, Dujardin A, Ortega N, Girard J-P (2012). Endogenous IL-33 is highly expressed in mouse epithelial barrier tissues, lymphoid organs, brain, embryos, and inflamed tissues: in situ analysis using a novel Il-33-LacZ gene trap reporter strain. J Immunol.

[22] Monticelli LA, Osborne LC, Noti M, Tran SV, Zaiss DM, Artis D (2015). IL-33 promotes an innate immune pathway of intestinal tissue protection dependent on amphiregulin-EGFR interactions. Proc Natl Acad Sci.

[23] Pastorelli L, Garg RR, Hoang SB, Spina L, Mattioli B, Scarpa M, Fiocchi C, Vecchi M, Pizarro TT (2010). Epithelial-derived IL-33 and its receptor ST2 are dysregulated in ulcerative colitis and in experimental Th1/Th2 driven enteritis. Proc Natl Acad Sci.

[24] Sponheim J, Pollheimer J, Olsen T, Balogh J, Hammarström C, Loos T, Kasprzycka M, Sørensen DR, Nilsen HR, Küchler AM (2010). Inflammatory bowel disease-associated interleukin-33 is preferentially expressed in ulceration-associated myofibroblasts. Am J Pathol.

[25] Ajduković J, Tonkić A, Salamunić I, Hozo I, Simunić M, Bonacin D (2010). Interleukins IL-33 and IL-17/IL-A in patients with ulcerative colitis. Hepatogastroenterology.

[26] Komai Koma M, Xu D, Li Y, McKenzie AN, McInnes IB, Liew FY (2007). IL-33 is a chemoattractant for human Th2 cells. Eur J Immunol.

[27] Schmitz J, Owyang A, Oldham E, Song Y, Murphy E, McClanahan TK, Zurawski G, Moshrefi M, Qin J, Li X (2005). IL-33, an interleukin-1-like cytokine that signals via the IL-1 receptor-related protein ST2 and induces T helper type 2-associated cytokines. Immunity.

[28] Seo DH, Che X, Kwak MS, Kim S, Kim JH, Ma HW, Kim DH, Kim TI, Kim WH, Kim SW (2017). Interleukin33 regulates intestinal inflammation by modulating macrophages in inflammatory bowel disease. Sci Rep.

[29] Kobori A, Yagi Y, Imaeda H, Ban H, Bamba S, Tsujikawa T, Saito Y, Fujiyama Y, Andoh A (2010). Interleukin-33 expression is specifically enhanced in inflamed mucosa of ulcerative colitis. J Gastroenterol.

[30] Fuss IJ (2008). Is the Th1/Th2 paradigm of immune regulation applicable to IBD?. Inflamm Bowel Dis.

[31] Park JH, Jeong DY, Peyrin-Biroulet L, Eisenhut M, Shin JI (2017). Insight into the role of TSLP in inflammatory bowel diseases. Autoimmun Rev.

[32] Beltrán CJ, Núñez LE, Díaz Jiménez D, Farfan N, Candia E, Heine C, Lopez F, González MJ, Quera R, Hermoso MA (2010). Characterization of the novel ST2/IL33 system in patients with inflammatory bowel disease. Inflamm Bowel Dis.

